# Time-resolved targeted metabolomics shows an abrupt switch from Calvin-Benson-Bassham cycle to tricarboxylic acid cycle when the light is turned off

**DOI:** 10.1007/s11120-025-01173-2

**Published:** 2025-09-29

**Authors:** Yuan Xu, Stephanie C. Schmiege, Thomas D. Sharkey

**Affiliations:** 1 MSU-DOE Plant Research Laboratory, Michigan State University, East Lansing, MI 48824 USA; 2 Plant Resilience Institute, Michigan State University, East Lansing, MI 48824 USA; 3 Department of Biology, Western University, London, ON N6A 5B7 Canada; 4 Department of Biological Sciences, Union College Schenectady, New York, 12308 USA; 5 Department of Biochemistry and Molecular Biology, Michigan State University, East Lansing, MI 48824 USA

**Keywords:** Calvin Benson Bassham cycle, Light transition, Photosynthetic carbon metabolism, Respiration, Tricarboxylic acid cycle

## Abstract

In leaves, major CO₂ fluxes alternate between fixation by the Calvin-Benson-Bassham (CBB) cycle during light and release by the tricarboxylic acid (TCA) cycle in darkness. The speed at which leaf metabolism transitions between these pathways likely influences plant tolerance to fluctuating light conditions. To investigate these rapid metabolic shifts, we exposed leaves to ¹³CO₂ for 20 min to establish a quasi-steady state before abruptly turning off the light while maintaining ¹³CO₂ feeding. Within 10 s of dark transition, 3-phosphoglycerate levels rose significantly while most other CBB cycle intermediates decreased by more than 90%. Simultaneously, carbon accumulated in alanine, likely via pyruvate. Over the subsequent 10 min, six- and five-carbon TCA cycle intermediates steadily increased. In contrast, four-carbon TCA intermediates peaked at one minute, declined at three minutes, and rose again at 10 min, a pattern mirrored by most measured amino acids. These results reveal an exceptionally rapid metabolic reconfiguration from CO₂ fixation by the CBB cycle in light to TCA cycle activation for energy production in darkness, accompanied by substantial changes in amino acid metabolism.

**Supplementary Information:**

The online version contains supplementary material available at 10.1007/s11120-025-01173-2.

## Introduction

Plant leaves use energy in sunlight for metabolic needs and carbon fixation by the Calvin-Benson-Bassham (CBB) cycle in the light. In the dark they rely on energy from glycolysis and the tricarboxylic acid (TCA). A significant amount of carbon fixed using sunlight energy during the day is stored as starch, which is then broken down at night to supply sugars for glycolysis and the TCA cycle. The speed with which the CBB cycle stops and the TCA cycle begins is unknown but may be important for understanding how carbon metabolism responds to fluctuating light.

Light fluctuations can occur over very short time periods, for example during leaf flutter of trembling aspen (Roden and Pearcy 1993) or over much longer periods, for example leaves at the bottom of the canopy (Shao et al. 2024; Durand et al. 2022) of trees and crop species. During the shortest light flecks, photosynthetic electron transport could be greatly affected (De Souza et al. 2022). The pools of the metabolites of the CBB cycle are small, most compounds have half-lives of 1 s or less (Szecowka et al. 2013; Arrivault et al. 2009) and so even short light flecks may have a big influence on carbon metabolism and the activity of the CBB and TCA cycles.

It has been assumed that ribulose 1,5-bisphosphate (RuBP) production ceases immediately when a leaf is exposed to darkness. In fact post illumination CO_2_ uptake has been taken as a measure of the pool of RuBP present when the light is turned off (Laisk et al. 1984). The need for reducing power traps metabolites of the CBB cycle in 3-phosphoglycerate (PGA). This requires regulation of downstream reactions so that metabolism can be reoriented from CBB cycle to heterotrophy and the TCA cycle.

These downstream reactions include lower glycolysis (from triose phosphates to pyruvate), which does not occur in chloroplasts (Stitt et al. 1978; Evans et al. 2024) preventing futile cycling. There is good evidence that the mitochondrial pyruvate dehydrogenase complex (mtPDC) is “inactivated by phosphorylation” in the light (Budde and Randall 1990). The chloroplast PDC is not inhibited allowing for fatty acid synthesis. While fatty acid synthesis is essential to the plant metabolic flux analysis indicates that it occurs at a low rate not likely to influence carbon metabolism rate in the light (Xu et al. 2022). Although a very small flow of recently fixed ^13^CO_2_ from citrate to glutamine and glutamate by way of α-ketoglutarate can be detected, for the four-carbon members of the TCA cycle, labeling is below the limits of detection (Xu et al. 2022; Szecowka et al. 2013). The near total lack of activity of the TCA cycle in the light has been known for many years. Calvin and Massini (1952) saw a very rapid increase in label in citrate upon darkness. The small flux toward α-ketoglutarate in the light may allow stored citrate to contribute to nitrogen metabolism (Gauthier et al. 2010; Abadie et al. 2017). Recently it has been recognized that, during photorespiration, serine metabolism associated with photorespiration may play a large role in amino acid metabolism (Fu et al. 2023; Busch et al. 2018).

In this study, we used time-resolved, targeted metabolomics to explore how carbon moves through metabolites during a transition from the CBB cycle to the TCA cycle, and how this transition influences energy metabolism and nitrogen assimilation. Unraveling these mechanisms is essential for understanding how plants maintain metabolic homeostasis and optimize energy utilization in dynamic light environments. The availability of stable isotopes (specifically ^13^CO_2_) provides a mechanism for following carbon when it is fed as ^13^CO_2_ to photosynthesizing leaves. This avoids potential complications when molecules are fed through the transpiration stream.

Here we report on measurements in which ^13^CO_2_ was fed to a photosynthesizing leaf for 20 min to achieve a quasi-steady-state (Xu et al. 2025) then abruptly put into darkness. We predicted that the change from CBB To TCA cycle would occur quickly because:

1. Citrate and other TCA metabolites exhibit almost no labeling in the light but a very rapid rise in citrate labeling within s of turning the light off (Calvin and Massini 1952; Szecowka et al. 2013; Ma et al. 2014; Xu et al. 2022).
2. The metabolite pools of the CBB cycle are known to have very short half-lives (Arrivault et al. 2009; Badger et al. 1984; Hasunuma et al. 2010).

We relied on metabolic flux analysis that identified a time frame of 20 min as a semi steady state (Xu et al. 2022). Our intent was to resolve changes that occur in leaves over the first 10 min of darkness. Our results show a very rapid decline in chloroplast CBB cycle carbon metabolism and a slower increase in mitochondria TCA metabolism.

## Methods

### Plant growth

Tobacco (*Nicotiana tabacum*, ‘Samsun’) plants transformed with *Populus alba* isoprene synthase (ISPS) were provided by Claudia Vickers from the University of Queensland. Details regarding plasmid design, vector construction, transformation, and line selection are described in Vickers et al. (2009). The IE transgenic lines used in this study resist insect pressure and showed normal morphological development and growth characteristics comparable to wild-type plants. No significant differences were observed in primary metabolic indicators, including photosynthetic pigment content and overall plant vigor, confirming that isoprene synthase expression did not adversely affect fundamental metabolic processes (Vickers et al. 2009). Plants were cultivated in greenhouses at the Michigan State University Plant Research Laboratory. The plants were maintained under a 16-hour photoperiod with a light intensity of 400–500 µmol m⁻² s⁻¹ provided by sunlight supplemented by sodium vapor lamps. Day/night temperatures were set to 25–27 °C/20–22 °C. Seeds were sown in Suremix growing medium (Michigan Grower Products, Galesburg, MI, USA) on separate trays. Fourteen days post-germination, seedlings were transplanted into 3.5 L pots (five seedlings per pot) to ensure survival. Two weeks after transplantation, when seedlings were stable, they were transferred to 7 L pots (one plant per pot). Plants were watered with deionized water for the first two days, followed by one-half-strength Hoagland’s nutrient solution (Hoagland and Arnon 1938) for all subsequent days. Experiments were conducted on 6- to 8-week-old plants before flowering and seed development. Three biological replicates were used.

### Sampling method

We employed an in-house-modified Fast Kill freeze clamp, as described by Sharkey et al. (2020), for this study. The setup integrated a LI-COR 6800 head with a 6 cm × 6 cm chamber. The chamber was sealed using cling film to create a closed environment and uniformly illuminated with four gooseneck fiber optic illuminators fed by two Schott KL2500 LED lamps, obtained from Edmund Optics, https://www.edmundoptics.com. The photosynthetic photon flux density was 1,000 µmol m⁻² s⁻¹. Chamber temperature was controlled and monitored using a thermocouple connected to the LI-6800 portable gas exchange system. The LI-COR 6800 instrument was configured to scrub all CO₂ from the system before introducing CO₂ from one of two sources: 5% ¹²CO₂ in air with natural isotopic abundance from Airgas (www.airgas.com) or a lecture bottle containing 99+% ¹³CO₂ (Aldrich, Sigma-Aldrich.com) pressurized with N₂. Flow meters (Alicat Scientific) regulated the delivery of ¹²CO₂ and ¹³CO₂. To achieve the desired CO₂ concentration, the ¹²CO₂ flow rate was adjusted based on the ¹³CO₂ concentration in the pressurized tank, using flow meter settings. The two flow meters were connected to the air supply entering the LI-COR 6800 head via a four-way valve. Leaves were equilibrated in the chamber under illumination for 30–60 min until assimilation rates stabilized. At this point, the CO₂ source was switched to ¹³CO₂ and maintained for 20 min, corresponding to the second of three labeling phases (Xu et al. 2022). The ¹³CO₂ supply was regulated to maintain a consistent CO₂ concentration of 420 ppm, and the transition from ¹²CO₂ to ~ 90% ¹³CO₂ was achieved within one minute. After 20 min, the light was turned off, and the plants were kept in darkness under black light-blocking cloth for varying durations (0, 10, 30, 60, 180, or 600 s). The Fast Kill mechanism was then triggered, using liquid-nitrogen-cooled copper blocks to rapidly freeze leaf samples. The interval between light interruption and the leaf sample reaching a temperature below 0 °C was measured at 35 ms (Sahu et al. 2023). Frozen leaf samples were collected in 2 mL microcentrifuge tubes and stored at − 80 °C for subsequent mass spectrometry analysis.

### Mass spectrometry

Metabolites were extracted from flash-frozen tissues according to established protocols (Xu et al. 2021). Subsequent analyses were conducted via mass spectrometry, as detailed in previous reports (Xu et al. 2021, 2022). Specific MS parameters for multiple reaction monitoring (MRM) with LC–MS/MS, selected ion monitoring (SIM), and GC–MS are provided in Supplemental Table S1. Phosphorylated metabolites in the CBB cycle were analyzed using ion-pair chromatography–tandem mass spectrometry (IPC–MS/MS). Separation was performed on a 2.1 × 50 mm ACQUITY UPLC BEH C18 column (Waters, Milford, MA, USA) connected to an ACQUITY UPLC pump system coupled with a Waters XEVO TQ-S UPLC/MS/MS (Waters, Milford, MA, USA).

Nucleotide sugars and other phosphorylated intermediates were measured by anion exchange chromatography–tandem mass spectrometry (AEC–MS/MS). Samples were run on a 2 × 250 mm IonPac AS11 analytical column with a 2 × 50 mm IonPac AG11 guard column (Dionex), using an ACQUITY UPLC pump system (Waters, Milford, MA, USA) coupled to a Xevo ACQUITY TQ Triple Quadrupole Detector (Waters, Milford, MA, USA). A self-regenerating suppressor (Dionex ADRS 600, Thermo Scientific, Waltham, MA, USA) was employed post-column to neutralize the KOH eluent.

Amino acids and organic acids were examined by GC–MS using an Agilent 7890 GC system interfaced with an Agilent 5975 C inert XL Mass Selective Detector (Agilent, Santa Clara, CA, USA). Initial derivatization with methoxyamine hydrochloride in dry pyridine was followed by silylation of amino and organic acids to form TBDMS derivatives (using N-(tertbutyldimethylsilyl)-N-methyltrifluoroacetamide with 1% [w/v] tert-butyldimethylchlorosilane). Separation was achieved on an Agilent VF5ms GC column (Agilent, Santa Clara, CA, USA).

Mass spectrometry data were analyzed to quantify pool size, mass isotopologue distributions (MIDs), and ¹³C enrichment, following previously described methods (Xu et al. 2022). LC–MS/MS data were acquired using MassLynx 4.0 (Agilent, Santa Clara, CA, USA), while GC–MS data were obtained with Agilent GC/MSD Chemstation (Agilent, Santa Clara, CA, USA). Peak detection and quantification were performed using the TargetLynx Application Manager within Waters MassLynx™ Software (Waters Corporation, MA). MIDs for GC–MS–measured metabolites were corrected for natural isotopic abundance using FluxFix (Trefely et al. 2016).

## Results

### Calvin-Benson-Bassham cycle declines when the light is turned off

We distinguish among three measures of each metabolite-.

- the total amount of the metabolite, nmol metabolite g^− 1^ FW.
- the relative degree of label incorporation; 0%, all carbons are ^12^C; 100%, all carbons are ^13^C.
- the amount of ^13^C atoms in each metabolite pool, nmol ^13^C g^− 1^ FW (= a times b times number of carbons in the molecule).

**Fig. 1:**
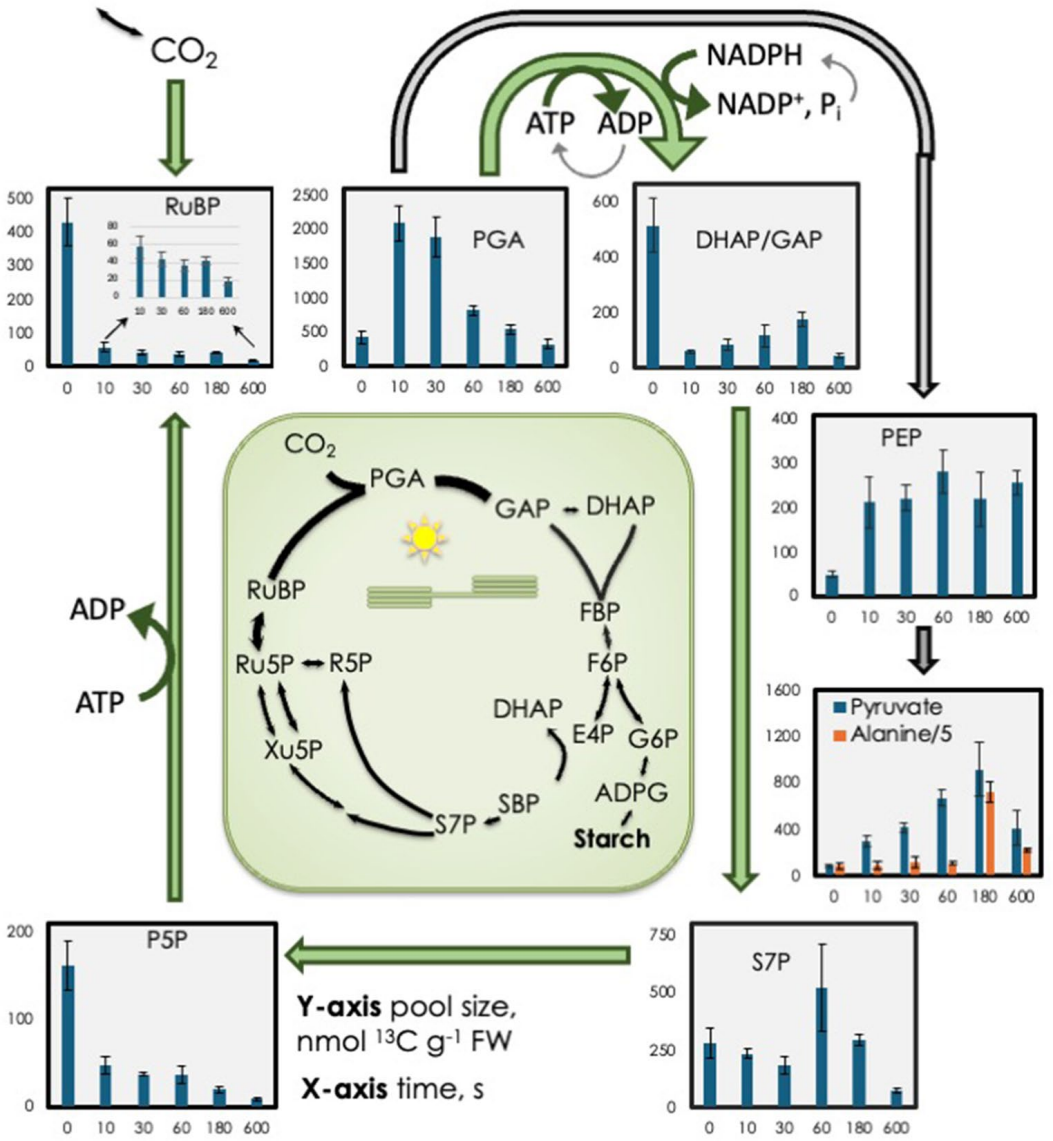
^13^C content of selected Calvin-Benson-Bassham (CBB) cycle intermediates and phosphoenolpyruvate, pyruvate, and alanine. Alanine values were divided by 5 so that they could fit on the same scale as pyruvate. The data are arranged to show the carbon flow from one metabolite to the next. Leaves were fed 420 ppm of ^13^CO_2_ for 20 min. At time 0 the light was turned off and pool sizes of various metabolites were measured for 10 min (600 s). Blue arrows indicate CBB cycle fluxes while grey arrows denote metabolism that is stimulated for the first 3 min of darkness. Bars show mean of three biological replicates ± standard deviation. RuBP = ribulose 1,5-bisphosphate, PGA = 3-phosphoglycerate, DHAP = dihydroxyacetone phosphate, GAP = glyceraldehyde 3-phosphate, PEP = phosphoenolpyruvate, S7P = sedoheptulose 7-phosphate, P5P = pentose phosphates

When the light was abruptly turned off the amount of ribulose 1,5-bisphosphate (RuBP) fell by almost 90% at the first time point of 10 s (Fig. 1). Other metabolites of the CBB cycle also fell except for PGA and sedoheptulose 7-phosphate (S7P). The increase in PGA was four-fold greater than the decrease in RuBP even though only a two-fold increase in PGA would be expected because of RuBP carboxylation. This indicates that pools other than RuBP contributed to the PGA accumulation. The S7P data differs from other CBB cycle intermediates. This is often observed (Xu et al. 2024) and may be related to a pool of sedoheptulose 1,7-bisphosphate (SBP) in the cytosol. E4P can cross the chloroplast membrane via the xylulose 5-phosphate/phosphate transporter (Hilgers et al. 2018), and cytosolic aldolase can combine this E4P with GAP to form SBP in the cytosol. However, this cytosolic SBP represents a metabolically inert pool because SBPase is absent from the cytosol. Cytosolic SBP can slowly equilibrate with the stromal SBP pool, causing whole-leaf measurements of SBP enrichment to be diluted by the unlabeled cytosolic pool, while S7P measurements reflect a mix of stromal metabolites and the slowly equilibrating SBP. This compartmentalization effect makes S7P labeling kinetics distinct from other CBB cycle intermediates, justifying its exclusion from our analyses of CBB cycle dynamics.

**Fig. 2:**
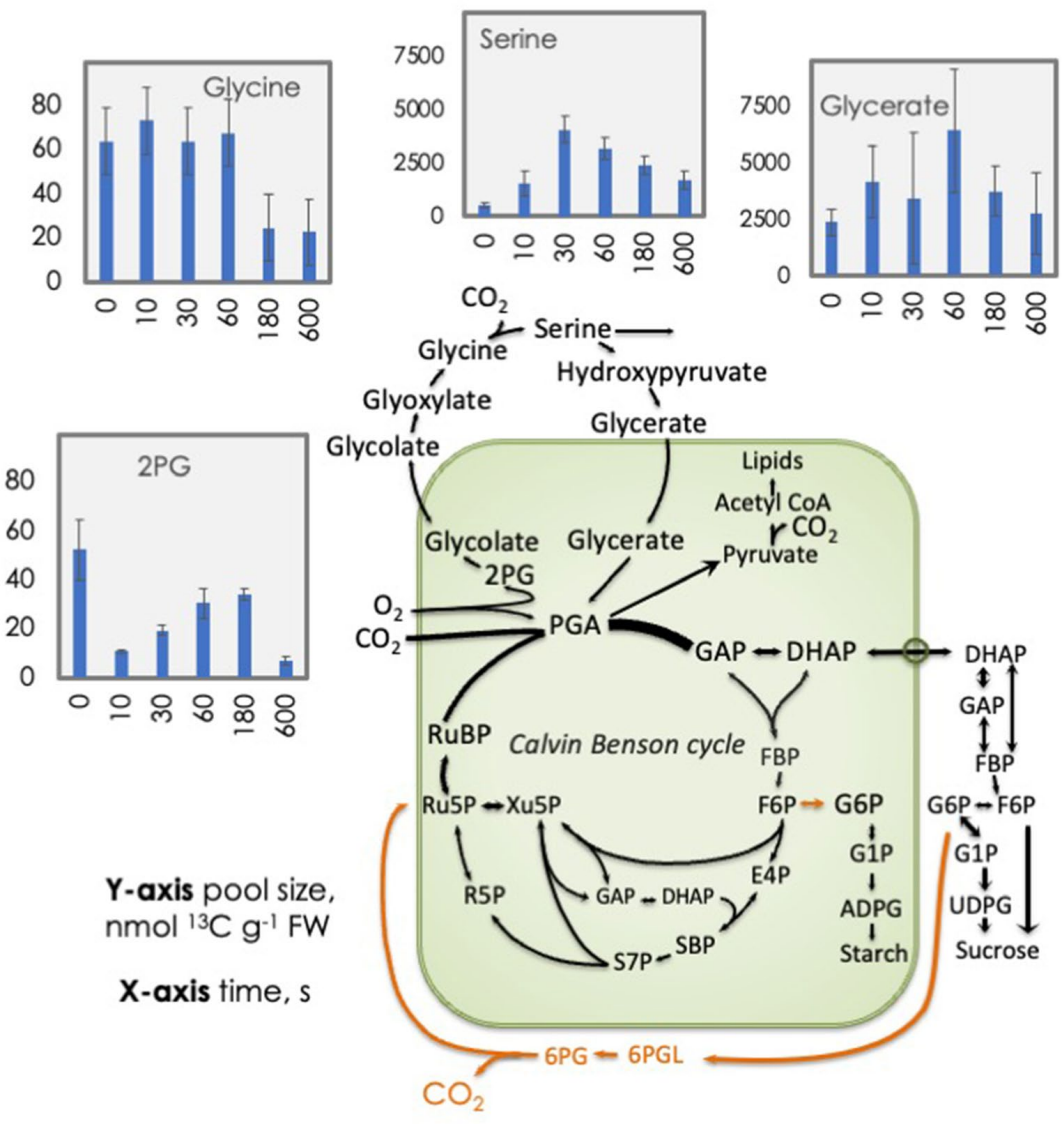
^13^C content of selected photorespiratory cycle intermediates. Leaves were fed 420 ppm of ^13^CO_2_ for 20 min. At time 0 the light was turned off ^13^C contents of various metabolites were measured for 10 min (600 s). RuBP = ribulose 1,5-bisphosphate, PGA = 3-phosphoglycerate, DHAP = dihydroxyacetone phosphate, GAP = glyceraldehyde 3-phosphate, PEP = phospho*enol*pyruvate, 2-PG = 2-phosphoglycoltae, S7P = sedoheptulose 7-phosphate, P5P = pentose phosphates. Orange shows the oxidative pentose phosphate pathway that could contribute to the post-illumination burst of CO_2_

The photorespiratory intermediate 2-phosphglycolate (2-PG) fell significantly in the first 10 s, mirroring the change in RuBP (Fig. 2). Glycine fell at 3 and 10 min but was stable up to 1 min. Serine and glycerate were present at much higher levels than 2PG and glycine (note the scales in Fig. 2).

**Fig. 3:**
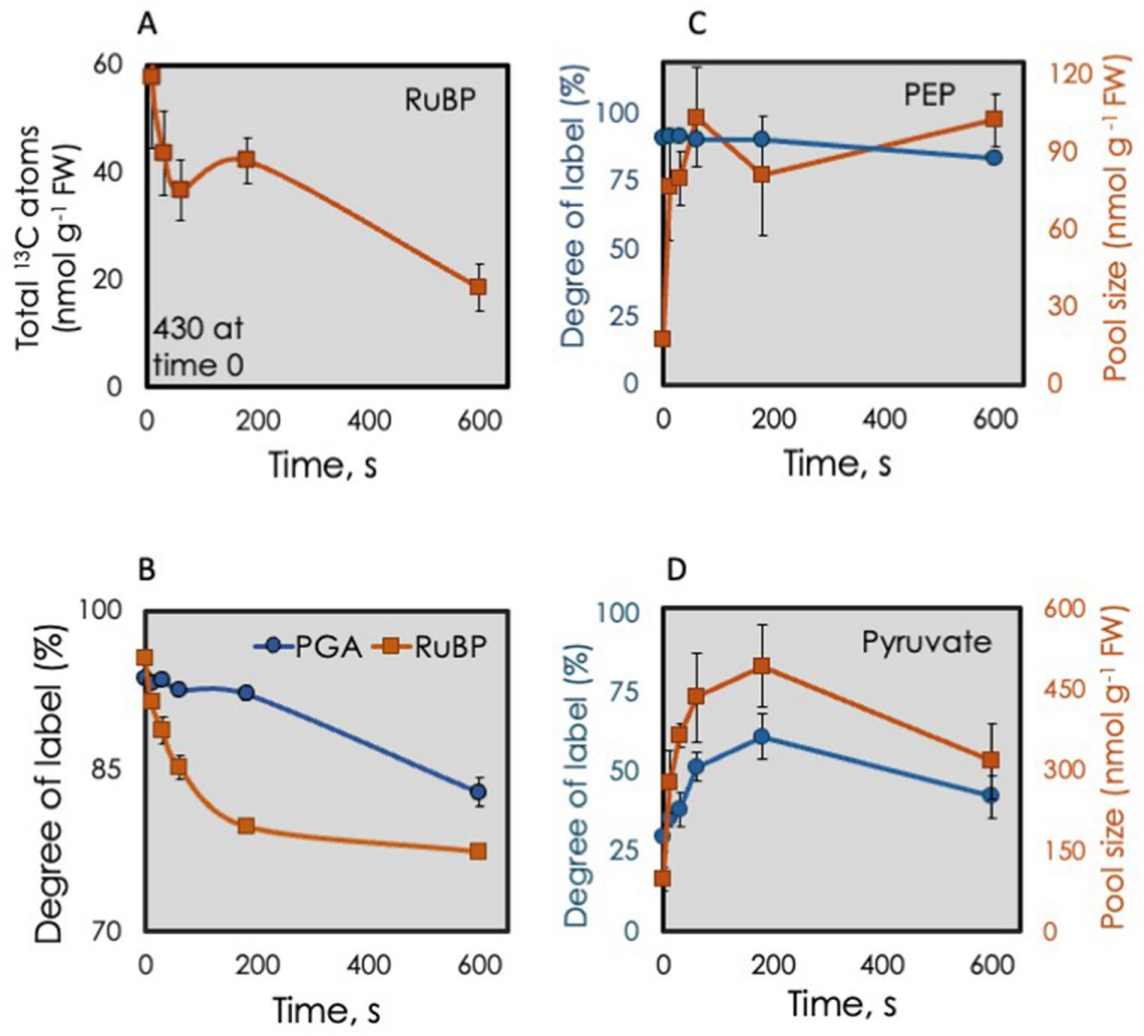
^13^C contents and total amounts of selected photosynthetic intermediates. Leaves were fed 420 ppm of ^13^CO_2_ for 20 min. At time 0 the light was turned off. **A**, ^13^C content of ribulose 1,5-bisphosphate (the same data as in Fig. 1 but plotted differently). **B**, Degree of label of RuBP (orange squares) and PGA (blue circles). **C**, Degree of label (blue circles) and pool size (orange squares, from Fig. 1) of PEP = (phosphoenolpyruvate). **D**, Degree of label of pyruvate (blue circles) and pool size (orange squares)

The amount of ^13^C in RuBP (pool size times degree of label) fell 87% in the first 10 s of darkness (Fig. 3A). This decline was similar to the decline in RuBP content (Fig. 1), which was reflected in a very small change in the degree of label in RuBP (Fig. 3B). The degree of label in RuBP and PGA (%^13^C) was similar at time zero (Fig. 3B). Over 10 min, the percentage of carbon atoms that were ^13^C in RuBP fell from 95% to 77% but the degree of label was noticeably higher in PGA (Fig. 3B). Any carboxylation that may have occurred in the dark would be supplying 99% ^13^CO_2_ to the one-sixth of PGA resulting from carboxylation of RuBP. We expect slow fluxes of unlabeled carbon to RuBP possibly through the cytosolic G6P shunt (Xu et al. 2024) and other metabolism of unlabeled molecules.

The initial increase in PGA (Fig. 1) coincided with an increase in PEP (Fig. 3C). A significant portion of the labeled carbon from 3-PGA was converted to pyruvate, with pyruvate ¹³C content increasing 3-fold by 3 min after darkening. Conversion of PGA to pyruvate inside the chloroplasts is negligible (Evans et al. 2024). Presumably PEP acts as an intermediate, which after the first 10 s, filling and emptying at rates that match, keeping the total pool size constant. The amount of PEP increased initially while the degree of label remained high (Fig. 3C), indicating that the carbon in PEP came from carbon in the highly enriched CBB cycle intermediates. On the other hand, *label* in pyruvate was low in the light (zero time) but increased for three min. This increase in ^13^C was paralleled by an increase in total *amount* of pyruvate. (Fig. 3D).

### Transition from Calvin-Benson-Bassham cycle to the tricarboxylic acid cycle

Labeling of pyruvate was limited during photosynthesis (Fig. 3D) even though PEP was heavily labeled (Fig. 3C). The degree of label in pyruvate never exceeded 62% (Supplemental Table S2). The large increase in PEP for the first few s did not result in any dilution of the label in PEP indicating that the increase in PEP resulted from conversion of the active pool of PGA to PEP. This reaction sequence is kept low in the light given that the amount of PEP was low at time zero.

There appeared to be a large unlabeled pool of pyruvate. When darkness was imposed, both the amount and degree of label in pyruvate increased for 3 min and then fell (Fig. 1. and 3D). This indicates that much of the increase in pyruvate resulted from PEP conversion to pyruvate. Between 3 and 10 min, pyruvate declined. Citrate increased and the degree of increase in citrate exceeded the decline in ^13^C in pyruvate. By 10 min the amount of pyruvate declined as did the degree of label indicating that there is a large metabolically inert pool of pyruvate. It is known that a large amount of pyruvate is in the vacuole (Szecowka et al. 2013).

**Fig. 4:**
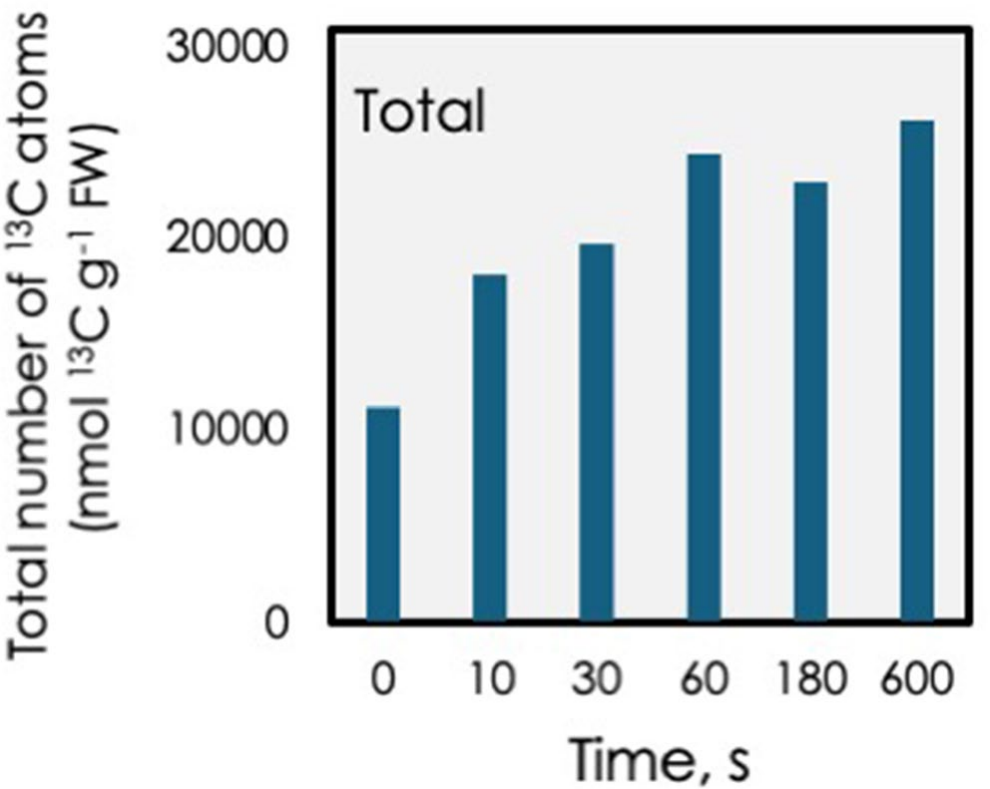
Number of ^13^C-labeled atoms in all metabolites measured

When the metabolites measured here were summed up, it appeared that the total ^13^C content increased significantly in the first 10 s and climbed at a slower rate for the next min (Fig. 4) but changed very little between 1 and 10 min. Post-illumination CO_2_ fixation attributable to carboxylation of RuBP (77.5 ^13^C nmol atoms g^− 1^ FW, Fig. 2 and supplemental Table S2) was not sufficient to account for the extra ^13^C atoms in citrate (12,398 ^13^C nmol atoms g^− 1^ FW). We presume that a more comprehensive accounting, and accounting for other metabolites being converted to RuBP, might explain the higher-than-expected ^13^C content of citrate.

### The tricarboxylic acid pathway response to darkness

**Fig. 5:**
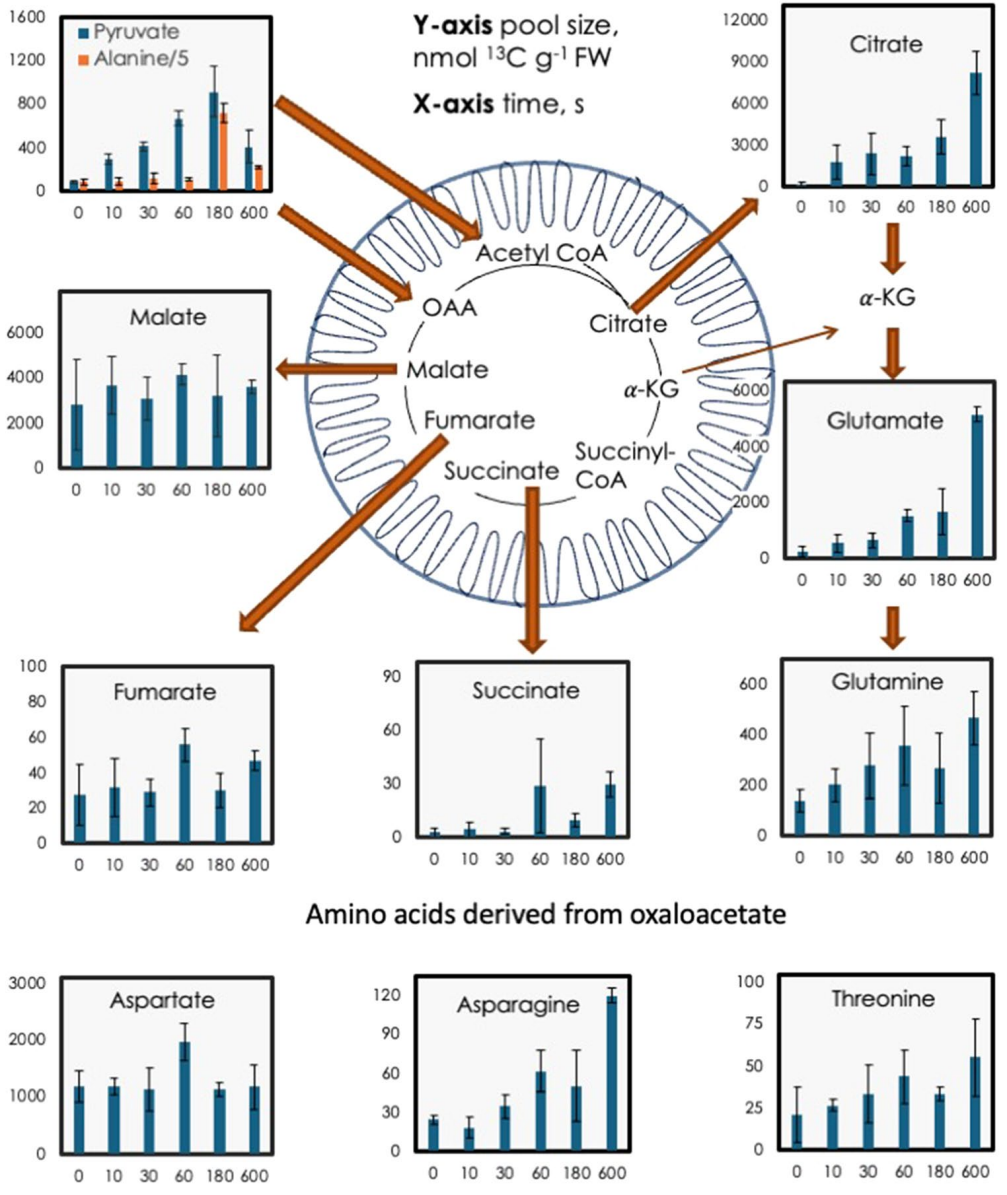
Pool sizes of selected tricarboxylic acid (TCA) cycle intermediates plus pyruvate, and alanine (from Fig. 1). Leaves were fed 420 ppm of ^13^CO_2_ for 20 min. At time 0 the light was turned off and pool sizes of various metabolites were measured over the next 10 min. Red arrows indicate TCA cycle fluxes. 𝛼-ketoglutarate (𝛼-KG) could not be measured but amino acids derived from 𝛼-KG, glutamate and glutamine, are shown. Similarly, amino acids made from oxaloacetate are shown. Data is the mean of three biological replicates ± standard deviation

**Fig. 6:**
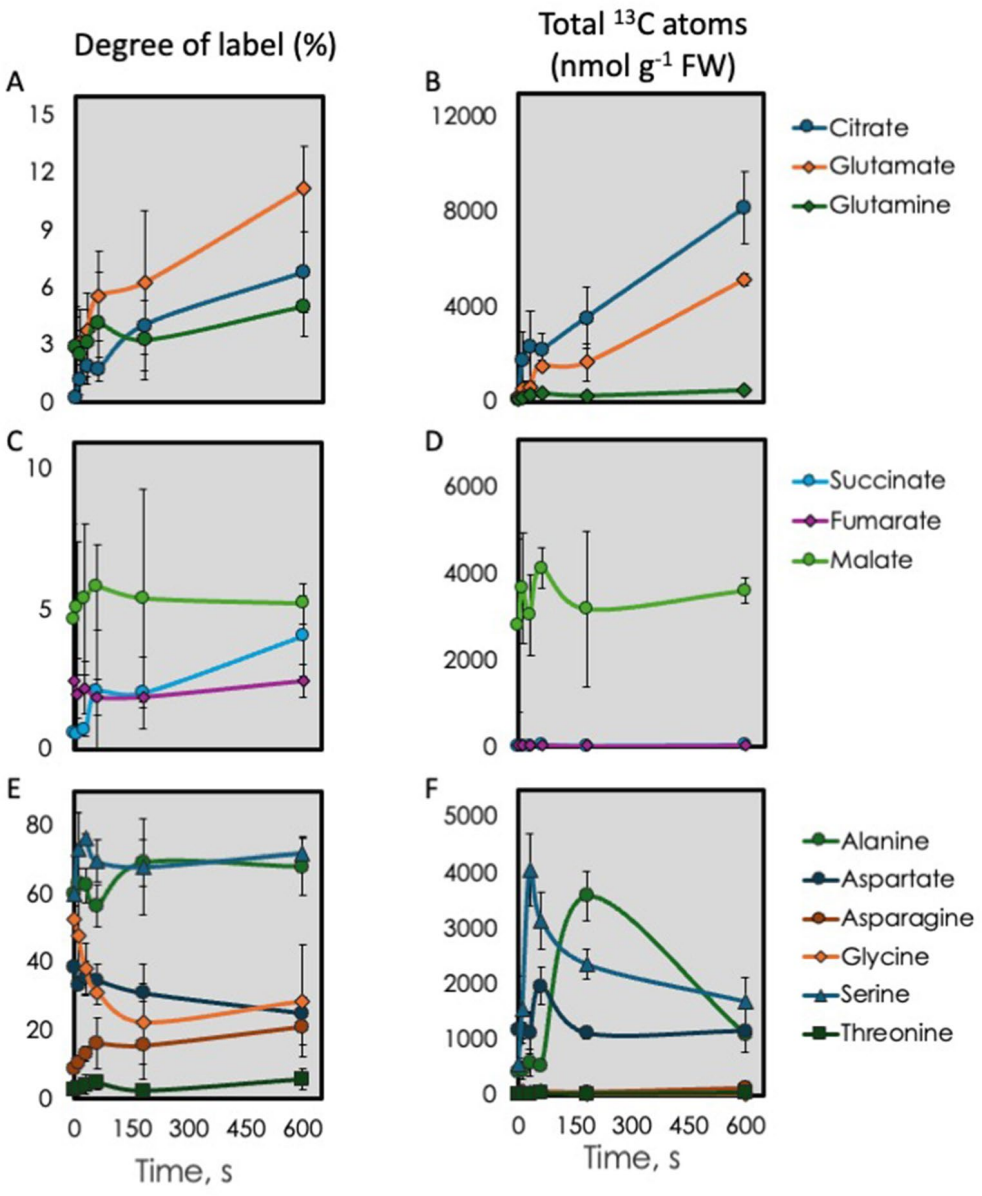
Degree of label and total ^13^C atoms in selected metabolites. **A**,** C**,** E**, Degree of label for citrate and 5-carbon amino acids, 4-carbon metabolites, and other amino acids respectively. **B**,** D**,** F**, ^13^C atoms in the metabolites

**Fig. 7:**
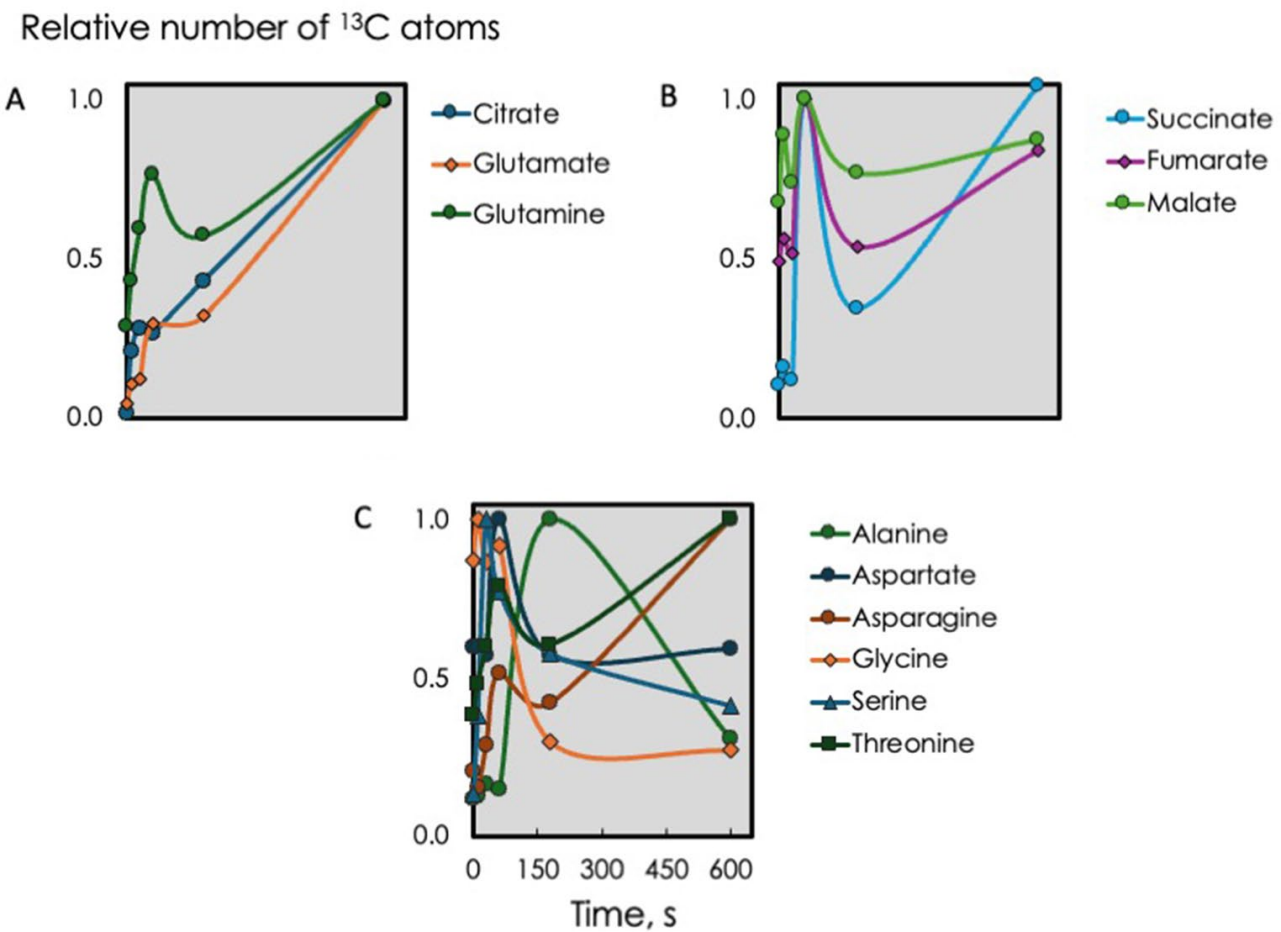
Relative number of ¹³C-labeled carbon atoms in the indicated metabolites, normalized to their respective maximum values

The amount of citrate jumped in the first 10 s but the additional ^13^C accounted for just 2% of this increase in citrate amount (Fig. 5 and Supplemental Table S2). Citrate, glutamate (as a proxy for α-ketoglutarate), and glutamine increased over the whole 10 min although glutamine, and to a lesser degree glutamate, did not increase as much as would be expected at 3 min (Fig. 5). These compounds had a low degree of label (Fig. 6A) and so most of the change in total content was from unlabeled sources, in line with Gauthier et al. (2010) and Abadie et al. (2017). The four-carbon metabolites succinate, fumarate and, to some degree malate, plus amino acids derived from oxaloacetate, exhibited a distinct peak at 1 min, then declined at 3 min but increased again at 10 min (Fig. 6 and Supplemental Table S2). The 4-carbon members of the TCA cycle, except malate, also had a low degree of label and low content of ^13^C (Fig. 6C and D). Malate labeling was higher than succinate and fumarate but was still low (Fig. 7C) This could indicate a slightly active PEP carboxylase. The low and relatively constant degree of label in succinate, fumarate, and malate resulted in little change to the amount ^13^C in these metabolites (Fig. 6C).

### Amino acids

The amount of glutamate increased over the 10 min of darkness, much like citrate (Fig. 5). On the other hand, changes in the amount of glutamine were more like other amino acids (Fig. 5). Alanine and serine were heavily labeled and remained so for 10 min (Fig. 6E), but aspartate, asparagine and threonine were relatively unlabeled (Fig. 7E). Unlike the *degree* of label, the *amount* of label in serine peaked at 30 s while there was an even larger pool of alanine at 3 min (Fig. 6F). This indicated that these pools filled with carbon from the CBB cycle and then emptied. The increase happened later for alanine but to a much greater extent (Fig. 6F). The large accumulation of nitrogen in the form of alanine at 3 min coincided with a dip in other amino acids (Fig. 5).

To look for patterns we plotted the amount of ^13^C atoms relative to the maximum for that metabolite (Fig. 7). Citrate and glutamate showed a steadily increasing relative amount of label while glutamine showed a temporary drop at 3 min (Fig. 7A), similar to most other amino acids. Succinate, fumarate, and malate all showed a dip at 3 min (Fig. 7B). Most of the amino acids had distinct minima at 3 min with the notable exception of alanine which had a very large peak in relative amount at 3 min (Fig. 7C). This was also seen in the total amount of ^13^C in alanine (Fig. 6F). The degree of label in alanine was high and did not change over time (Fig. 6E) indicating that the large increase in alanine came from other CBB cycle intermediates.

## Discussion

The focus of this study was the change in carbon metabolism as the CBB cycle stops and TCA cycle begins. Carbon metabolism in leaves can be complex and involve many different pathways; how light or darkness will affect these pathways is difficult to discern (Tcherkez et al. 2024). There are many changes in plant cell metabolism that can be detected over a longer time frame. For example Abadie et al. (2021) found as many as 4,500 metabolic features when different CO_2_ and O_2_ levels were imposed on sunflower leaves for two hours followed by untargeted metabolomics. Dellero et al. (2024) found significant metabolic differences when leaves or leaf discs were fed ^13^C-glucose for 30 min to 6 h. The focus of the work reported here was on initial events in the switch from CBB cycle to TCA cycle using ^13^CO_2_ and a targeted metabolic approach. Our study does not address longer term changes such as gene expression or translation effects.

### Postillumination burst and light enhanced dark respiration

We did not measure gas exchange, but our results provided some insight into the metabolism changes during the post-illumination burst and light enhanced dark respiration. The post illumination burst (PIB) of CO_2_ thought to originate in photorespiratory metabolism (Gregory et al. 2024; Decker 1955; Rawsthorne and Hylton 1991) as 2-PG is converted to glycine which is then decarboxylated. We measured both 2-PG and glycine. The amount of glycine did not decline but in fact increased over time (Table S2). The amount of 2-PG declined significantly (two tailed T-test *p* = 0.003) from 27.1 ± 6.8 to 5.8 ± 0.7 nmol metabolites g^− 1^ FW over the first 10 s. It then recovered slightly but remained below the value in the light. However, glycine increased after 10 s and remained above the level in the light until 3 min after darkening. Thus, we did not see behavior of either 2-PG nor glycine that would be expected if the post-illumination burst was fed by either of these compounds, but it is possible that other metabolites were converted to glycine supporting the burst. It is also possible that the oxidative pentose phosphate pathway was operative in either the plastid or cytosol, providing CO_2_. The plastidial glucose-6-phosphate dehydrogenase is normally off in the light but could be activated quickly in the dark (Preiser et al. 2019). While we could not distinguish between glucose- and fructose 6-phosphate, nor between plastidial and cytosolic pools, we note that hexose phosphates, the substrate for the oxidative pentose phosphate pathways, declined monotonically by 30 nmol g^− 1^ FW by 10 min while for 2-PG the decline was just 21 nmol g^− 1^ FW at 10 s after which it recovered (Supplemental Table S2).

Another behavior seen when the light is turned off is a stimulation of oxygen uptake called light enhanced dark respiration. This is more common in bacteria and algae than plants (Shimakawa et al. 2020). It is not clear whether the metabolite changes reported here provide insight into the oxygen exchange at a light to dark transition indicative of light enhanced dark respiration.

### ATP/NADPH dynamics

In the light, high ATP/ADP and NADPH/NADP^+^ ratios enforce metabolism to proceed from PGA to triose phosphates. Our data suggests that reducing power has more effect than ATP in limiting the CBB cycle when light is first reduced. There are several lines of regarding the role of redox power limitation when darkness is imposed.

A. The ATP turn over time is slower than NADPH turnover time (Szecowka et al. 2013; Arrivault et al. 2009) (turnover time will reflect pool size divided by rate of metabolism through that metabolite).
B. Chlorophyll fluorescence studies indicate that some back reaction, from triose phosphates to PGA, can occur (light grey arrows in Fig. 1) providing some ATP. This can give rise to a transient in chlorophyll fluorescence that is affected by the presence or absence of fructose bisphosphate aldolase and the triose phosphate-phosphate transporter (Gotoh et al. 2010a). This behavior has been studied to learn about the pathways for cyclic electron flow (Gotoh et al. 2010b).
C. Jagendorf and Uribe (1966) and Sharkey et al. (1986) showed that there can be post-illumination ATP synthesis from the stored proton motive force gradient across the thylakoid membrane. In addition, conversion of PEP to pyruvate (plus alanine) could supply ATP by substrate phosphorylation. Mitochondrial electron transport activity could also supply ATP at the expense of reducing power supplying ATP for cytosolic needs, but this ATP is unlikely to enter the chloroplast (Voon and Lim 2019).

The rapid changes in ATP/NADPH ratios highlight the critical role of energy balance in regulating metabolic transitions (Hoefnagel et al. 1998). These findings suggest that plants have evolved precise mechanisms that regulate metabolite levels, and which maintain energy homeostasis during light-dark transitions, ensuring survival in stochastic light environments, such as forest canopies or dense crop fields.

The largest initial increase of metabolites of the CBB cycle after entering darkness, among those we measured, was PGA. The accumulation of PGA is advantageous since it can refill the CBB cycle without delay efficiently absorb high levels of light if the leaf is reilluminated after one min or less (Sharkey et al. 1986; Stitt 1986). However, after 10 min of darkness the PGA pool dissipates and so would not be available for refilling the CBB cycle (Fig. 1). Oxygen evolution can exhibit a greater-than-steady-state rate upon reillumination of a leaf while the excess PGA is reduced to triose phosphates (Stitt 1986; Kirschbaum and Pearcy 1988; Sharkey et al. 1986).

The metabolism from triose phosphates to pentose phosphates require no reducing power nor ATP. Because there are sources of ATP, pentoses produced after the light is off can be phosphorylated to RuBP and then carboxylated providing a highly labeled input into the PGA pool (rubisco and phosphoribulokinase can remain at least partially active for many minutes after switching to low light (Sassenrath-Cole and Pearcy 1994). We saw evidence for this as both RuBP and the pentose phosphate pool continued to decline for 10 min after the light was off. The significant decline in RuBP in the first 10 s was followed by a further small but measurable decline out to 10 min (Fig. 3A).

By 30 s the PGA pool began to fall; PEP increased at 10 s but then was constant while pyruvate, and then alanine, increased over 10 min. A large pool of PGA allows very rapid resumption of photosynthesis once reducing power (NADPH) becomes available but when the carbon moves on to pyruvate it becomes more difficult to repopulate the CBB cycle to restart the cycle. One mechanism that has been proposed for restarting the CBB cycle is a cytosolic shunt involving the oxidative pentose phosphate pathway (Xu et al. 2024). This would inject carbon as ribulose 5-phosphate that, because there is ATP available, would be easily converted to RuBP and so restart the cycle. This has been proposed for both plants (Xu et al. 2021) and cyanobacteria (Tanaka et al. 2022). C_4_ plants often have larger metabolite pools that could buffer the effects seen here and improve use of light flecks (Stitt and Zhu 2014).

We can describe three fates for PGA (Fig. 1). The normal route (green curved arrow) is for PGA to be converted to triose phosphates and eventually back to RuBP plus end products, usually mostly starch and sucrose. However, when there is no NADPH, PGA cannot be converted to triose phosphates. In this case, another pathway for PGA metabolism comes into play, conversion to PEP and then to pyruvate plus ADP (Fig. 1 light gray). The small amount of PEP at zero time (in the light) might indicate that the flow of carbon from PGA to PEP is normally low though the high degree of label indicates that carbon in PEP is in isotopic equilibrium with the CBB cycle. Within 10 s the amount of PEP increased and remained constant throughout the following 10 min. Because PGA was declining and pyruvate was increasing, the elevated but constant PEP may indicate significant but balanced synthesis and catabolism of PEP.

### Pyruvate

There was a large pool of unlabeled pyruvate in the light. In darkness total pyruvate increased further and the increase in pyruvate was labeled. The triose phosphate transporter exchanges 3- PGA for phosphate as efficiently as glyceraldehyde 3-phosphate and dihydroxyacetone phosphate (Flügge and Heldt 1991). Since triose phosphates and phosphate are largely doubly negatively charged, 2-, in physiological conditions while PGA can be 2- or 3-, transport of PGA will be relatively restricted by the pH gradient between the stroma and cytosol in the light but much less so in the dark. PGA exported in the dark could be converted to pyruvate. Pyruvate began to fall by 10 min and the degree of label fell, consistent with a large, metabolically inert, pool plus an active pool that occurred for three min following light off. Pyruvate kinase activity is catalyzed by any of a large number of pyruvate kinases with distinct kinetics and expression patterns (five cytosolic enzymes and a total of 14 in Arabidopsis) (Wulfert et al. 2020). Some pyruvate kinases are particularly susceptible to inhibition by ATP. Very high levels of PGA and relatively oxidized NAD^+^ could lead to PGA dehydrogenase activity (Krämer et al. 2024). This would provide some reducing power. Serine, a product of the pathway that begins with phosphoglycerate dehydrogenase, tripled in amount at 1 min darkness (Supplemental Table S2).

The lower part of glycolysis involves conversion of 3-PGA to 2-phosphoglycerate to PEP and then pyruvate. Because chloroplasts have little to no activity of enzymes in this pathway (Evans et al. 2024; Stitt and ap Rees 1979), the production of PEP and then pyruvate must occur in the cytosol of photosynthesizing leaves although some pyruvate can be made by rubisco (Evans et al. 2024). The amount of PEP in the leaf increased rapidly but the degree of label stayed the same and similar to CBB cycle intermediates (Fig. 3C). These findings suggest that PGA acts as a critical metabolic hub, enabling plants to efficiently manage carbon resources in dynamic light environments. This also suggests that pyruvate kinase in the cytosol is highly regulated and can become very active rapidly in the dark leading to an increase in heavily labeled pyruvate.

It is known that a large amount of pyruvate is in the vacuole (Szecowka et al. 2013). The mitochondrial pyruvate dehydrogenase complex is regulated; phosphorylation results in inactivation of the complex (Budde and Randall 1990). While some activity of the PDH complex may still occur in the light, the rate of labelling of citrate is exceedingly slow (Calvin and Massini 1952). The chloroplast pyruvate dehydrogenase complex is not regulated by phosphorylation and so can supply acetyl CoA from pyruvate for fatty acid synthesis etc. in the light (Camp and Randall 1985).

The TCA cycle appeared to increase in activity almost immediately. The amount of citrate increased from almost nothing as soon as 10 s after turning off the light (Fig. 5). The citrate and α-ketoglutarate increased monotonically for 10 min but the degree of label remained low (Table S2). The low labeling in most TCA cycle compounds indicates that rapid TCA cycle activation primarily utilizes stored carbon pools, while recently fixed carbon provides immediate energy and metabolic intermediates. Succinate, fumarate, and malate all showed a decrease in amount at 3 min and recovery at 10 min. This increase in TCA cycle intermediates would require anaplerotic reactions such as PEP carboxylase and activation of mitochondrial pyruvate dehydrogenase complex to provide acetyl CoA to make citrate. This could account for the declining pyruvate at min 10 and, if transaminases were active, the very large pool of alanine could feed the anaplerotic reactions. The rapid increase in pyruvate and TCA cycle activation demonstrates the metabolic flexibility of leaves in transitioning from CBB to TCA cycles, highlighting mitochondrial respiration as a critical energy source that is quickly activated when photosynthesis ceases.

### Nitrogen metabolism

Glutamine, aspartate, asparagine, and threonine all showed increases at 1 min but then a decrease in amount at 3 min and recovery at 10 min. Alanine was the notable exception, being very abundant at 3 min when nearly all other metabolites were down. Serine reached a maximum even earlier, at 30 s. This may indicate some role for alanine aminotransferases, a large family of enzymes (McAllister et al. 2013). Breakdown of alanine when it is in excess may be the primary function of alanine aminotransferases (Miyashita et al. 2007). Serine production during photorespiration has been invoked as a mechanism for supplying amino groups during photosynthesis (Busch et al. 2018; Fu et al. 2023); in the absence of photorespiration serine can be made by two other pathways both of which begin with PGA (Zimmermann et al. 2021; Igamberdiev and Kleczkowski 2018). It may be that the very high concentration of PGA at 10 and 30 s stimulated serine synthesis by one of these alternate mechanisms since the rate of carboxylation (and oxygenation) would be very low after 10 s but serine levels were highest at 30 s (Fig. 7C and supplemental Table S2). Significant changes over time, with amino groups on alanine increasing substantially at min 3 may reflect the adjustment of nitrogen metabolism from serine metabolism in the presence of photorespiration to GS-GOGAT directly during a light-dark transient. The shifts in nitrogen metabolism, particularly the accumulation of alanine and serine, highlight the tight coordination between carbon and nitrogen metabolism during light-dark transitions. These changes suggest that plants dynamically reallocate nitrogen resources to support metabolic reprogramming in response to light fluctuations, enhancing resilience and ensuring efficient resource use in dynamic light environments.

## Conclusion

Our results provide new insights into the carbon metabolism changes that occur when leaves transition from light to darkness. We show that the rapid depletion of CBB cycle intermediates and the activation of the TCA cycle enable a swift switch from chloroplast-based photosynthesis to mitochondrial respiration. In addition to the changes in carbon metabolism, nitrogen metabolism appears to undergo a large shift, highlighting their interconnectedness. This work adds another dimension to the studies of metabolism in a stochastic light environment, highlighting the metabolic flexibility of leaves and their ability to maintain homeostasis under changing light environments. This work examined a single step change from light to darkness, characterized by rapid shifts in carbon flow, energy production, and nitrogen assimilation. The ability to swiftly transition between photosynthetic and respiratory metabolic states provides plants with a competitive advantage in dynamic ecosystems, with potential significant implications for crop resilience and agricultural productivity. Future work might involve looking at low light availability, for example, what is the metabolic state near the light compensation point. Finally, this study may provide useful information for understanding carbon metabolism changes in leaves experiencing fluctuating light of different durations and may point to a role for changing nitrogen metabolism during light flecks.

## Supplementary Information

Below is the link to the electronic supplementary material.

Supplementary Material 1

## Data Availability

All the data are available in the main text and in the Supporting Information.

## References

[CR2] Abadie C, Lothier J, Boex-Fontvieille E, Carroll A, Tcherkez G (2017) Direct assessment of the metabolic origin of carbon atoms in glutamate from illuminated leaves using ^13^C-NMR. New Phytol 216(4):1079–1089. 10.1111/nph.14719 28771732 10.1111/nph.14719

[CR1] Abadie C, Lalande J, Limami AM, Tcherkez G (2021) Non-targeted ^13^C metabolite analysis demonstrates broad re‐orchestration of leaf metabolism when gas exchange conditions vary. Plant Cell Environ 44(2):445–457. 10.1111/pce.13940 33165970 10.1111/pce.13940

[CR3] Arrivault S, Guenther M, Ivakov A, Feil R, Vosloh D, van Dongen JT, Sulpice R, Stitt M (2009) Use of reverse-phase liquid chromatography, linked to tandem mass spectrometry, to profile the Calvin cycle and other metabolic intermediates in Arabidopsis rosettes at different carbon dioxide concentrations. Plant J 59(5):824–839. 10.1111/j.1365-313X.2009.03902.x 10.1111/j.1365-313X.2009.03902.x 19453453

[CR4] Badger MR, Sharkey TD, von Caemmerer S (1984) The relationship between steady-state gas exchange of bean leaves and the levels of carbon-reduction-cycle intermediates. Planta 160:305–313 24258579 10.1007/BF00393411

[CR5] Budde RJA, Randall DD (1990) Pea leaf mitochondrial pyruvate dehydrogenase complex is inactivated in vivo in a light-dependent manner. Proc Natl Acad Sci USA 87:673–676 11607058 10.1073/pnas.87.2.673 PMC53327

[CR6] Busch FA, Sage RF, Farquhar GD (2018) Plants increase CO_2_ uptake by assimilating nitrogen via the photorespiratory pathway. Nat Plants 4(1):46–54. 10.1038/s41477-017-0065-x 29229957 10.1038/s41477-017-0065-x

[CR7] Calvin M, Massini P (1952) The path of carbon in photosynthesis. XX. The steady state. Experientia 8(12):445–457 13021134 10.1007/BF02139287

[CR8] Camp PJ, Randall DD (1985) Purification and characterization of the pea chloroplast pyruvate dehydrogenase complex 1: A source of acetyl-CoA and NADH for fatty acid biosynthesis. Plant Physiol 77(3):571–577. 10.1104/pp.77.3.571 16664100 10.1104/pp.77.3.571 PMC1064566

[CR9] De Souza AP, Burgess SJ, Doran L, Hansen J, Manukyan L, Maryn N, Gotarkar D, Leonelli L, Niyogi KK, Long SP (2022) Soybean photosynthesis and crop yield are improved by accelerating recovery from photoprotection. Science 377(6608):851–854. 10.1126/science.adc9831 35981033 10.1126/science.adc9831

[CR10] Decker JP (1955) A rapid, postillumination deceleration of respiration in green leaves. Plant Physiol 30(1):82–84 16654735 10.1104/pp.30.1.82 PMC540603

[CR11] Dellero Y, Berardocco S, Bouchereau A (2024) U-^13^C-glucose incorporation into source leaves of brassica Napus highlights light-dependent regulations of metabolic fluxes within central carbon metabolism. J Plant Physiol 292:154162. 10.1016/j.jplph.2023.154162 38103478 10.1016/j.jplph.2023.154162

[CR12] Durand M, Stangl ZR, Salmon Y, Burgess AJ, Murchie EH, Robson TM (2022) Sunflecks in the upper canopy: dynamics of light-use efficiency in sun and shade leaves of *Fagus sylvatica*. New Phytol 235(4):1365–1378. 10.1111/nph.18222 35569099 10.1111/nph.18222 PMC9543657

[CR13] Evans SE, Xu Y, Bergman ME, Ford SA, Liu Y, Sharkey TD, Phillips MA (2024) Rubisco supplies pyruvate for the 2-C-methyl-D-erythritol-4-phosphate pathway. Nat Plants 10:1453–1463. 10.1038/s41477-024-01791-z 39367254 10.1038/s41477-024-01791-z

[CR14] Flügge UI, Heldt HW (1991) Metabolite translocators of the chloroplast envelope. Annu Rev Plant Physiol Plant Mol Biol 42:129–144

[CR15] Fu X, Gregory LM, Weise SE, Walker BJ (2023) Integrated flux and pool size analysis in plant central metabolism reveals unique roles of Glycine and Serine during photorespiration. Nat Plants 9(1):169–178. 10.1038/s41477-022-01294-9 36536013 10.1038/s41477-022-01294-9

[CR16] Gauthier PPG, Bligny R, Gout E, Mahé A, Nogués S, Hodges M, Tcherkez GGB (2010) In folio isotopic tracing demonstrates that nitrogen assimilation into glutamate is mostly independent from current CO_2_ assimilation in illuminated leaves of *Brassica Napus*. New Phytol 185(4):988–999. 10.1111/j.1469-8137.2009.03130.x 20070539 10.1111/j.1469-8137.2009.03130.x

[CR17] Gotoh E, Kobayashi Y, Tsuyama M (2010a) The post-illumination chlorophyll fluorescence transient indicates the rubp regeneration limitation of photosynthesis in low light in Arabidopsis. FEBS Lett 584(14):3061–3064. 10.1016/j.febslet.2010.05.039 20561988 10.1016/j.febslet.2010.05.039

[CR18] Gotoh E, Matsumoto M, Ogawa Ki, Kobayashi Y, Tsuyama M (2010b) A qualitative analysis of the regulation of cyclic electron flow around photosystem I from the post-illumination chlorophyll fluorescence transient in arabidopsis: a new platform for the in vivo investigation of the Chloroplast redox state. Photosynth Res 103(2):111–123. 10.1007/s11120-009-9525-0 20054711 10.1007/s11120-009-9525-0

[CR19] Gregory LM, Tejera-Nieves M, Walker BJ (2024) Measuring and quantifying characteristics of the post-illumination burst. In: Walker BJ (ed) Photorespiration: methods and protocols. Springer US, New York, NY, pp 115–124.10.1007/978-1-0716-3802-6_9 10.1007/978-1-0716-3802-6_9 38861082

[CR20] Hasunuma T, Harada K, Miyazawa S-I, Kondo A, Fukusaki E, Miyake C (2010) Metabolic turnover analysis by a combination of in vivo ^13^C-labelling from ^13^CO_2_ and metabolic profiling with CE-MS/MS reveals rate-limiting steps of the C_3_ photosynthetic pathway in *Nicotiana tabacum* leaves. J Exp Bot 61(4):1041–1051. 10.1093/jxb/erp374 20026474 10.1093/jxb/erp374 PMC2826653

[CR21] Hilgers EJA, Schöttler MA, Mettler-Altmann T, Krueger S, Dörmann P, Eicks M, Flügge U-I, Häusler RE (2018) The combined loss of triose phosphate and xylulose 5-phosphate/phosphate translocators leads to severe growth retardation and impaired photosynthesis in *Arabidopsis thaliana* tpt/xpt double mutants. Front Plant Sci 9(1331):1331. 10.3389/fpls.2018.01331 30333839 10.3389/fpls.2018.01331 PMC6175978

[CR22] Hoagland DR, Arnon DI (1938) The water culture method for growing plants without soil. In. UC Agric. Exp. Sta. Circular 347, Berkley, pp 1–39

[CR23] Hoefnagel MH, Atkin OK, Wiskich JT (1998) Interdependence between chloroplasts and mitochondria in the light and the dark. Biochim Et Biophys Acta: Bio-Energetics 1366(3):235–255

[CR24] Igamberdiev AU, Kleczkowski LA (2018) The glycerate and phosphorylated pathways of serine synthesis in plants: the branches of plant glycolysis linking carbon and nitrogen metabolism. Front Plant Sci 9. 10.3389/fpls.2018.00318 10.3389/fpls.2018.00318 PMC5861185 29593770

[CR25] Jagendorf AT, Uribe E (1966) ATP formation caused by acid-base transition of spinach chloroplasts. Proc Natl Acad Sci USA 55(1):170–177 5220864 10.1073/pnas.55.1.170 PMC285771

[CR26] Kirschbaum MUF, Pearcy RW (1988) Concurrent measurements of oxygen- and carbon-dioxide exchange during lightflecks in *Alocasia macrorrhiza* (L.) G. Don. Planta 174(4):527–533. 10.1007/BF00634483 24221570 10.1007/BF00634483

[CR27] Krämer M, Blanco NE, Penzler J-F, Davis GA, Brandt B, Leister D, Kunz H-H (2024) Cyclic electron flow compensates loss of PGDH3 and concomitant stromal NADH reduction. Sci Rep 14(1):29274. 10.1038/s41598-024-80836-x 39587304 10.1038/s41598-024-80836-x PMC11589868

[CR28] Laisk A, Kiirats O, Oja V (1984) Assimilatory power (postillumination CO_2_ uptake) in leaves: Measurement, environmental dependencies, and kinetic properties. Plant Physiol 76(3):723–729. 10.1104/pp.76.3.723 16663913 10.1104/pp.76.3.723 PMC1064362

[CR29] Ma F, Jazmin LJ, Young JD, Allen DK (2014) Isotopically nonstationary ^13^C flux analysis of changes in *Arabidopsis thaliana* leaf metabolism due to high light acclimation. Proc Natl Acad Sci USA 111(47):16967–16972. 10.1073/pnas.1319485111 25368168 10.1073/pnas.1319485111 PMC4250135

[CR30] McAllister CH, Facette M, Holt A, Good AG (2013) Analysis of the enzymatic properties of a broad family of alanine aminotransferases. PLoS ONE 8(2):e55032. 10.1371/journal.pone.0055032 23408955 10.1371/journal.pone.0055032 PMC3567105

[CR31] Miyashita Y, Dolferus R, Ismond KP, Good AG (2007) Alanine aminotransferase catalyses the breakdown of alanine after hypoxia in *Arabidopsis thaliana*. Plant J 49(6):1108–1121. 10.1111/j.1365-313X.2006.03023.x 17319845 10.1111/j.1365-313X.2006.03023.x

[CR32] Preiser AL, Fisher N, Banerjee A, Sharkey TD (2019) Plastidic glucose-6-phosphate dehydrogenases are regulated to maintain activity in the light. Biochem J 476(10):1539–1551. 10.1042/bcj20190234 31092702 10.1042/BCJ20190234 PMC6626494

[CR33] Rawsthorne S, Hylton CM (1991) The relationship between the post-illumination CO2 burst and glycine metabolism in leaves of C3 and C 3-C 4 intermediate species of Moricandia. Planta 186(1):122–126. 10.1007/bf00201507 24186584 10.1007/BF00201507

[CR34] Roden JS, Pearcy RW (1993) Effect of leaf flutter on the light environment of poplars. Oecologia 93(2):201–207. 10.1007/BF00317672 28313608 10.1007/BF00317672

[CR35] Sahu A, Mostofa MG, Weraduwage SM, Sharkey TD (2023) Hydroxymethylbutenyl diphosphate accumulation reveals MEP pathway regulation for high CO_2_-induced suppression of isoprene emission. Proceedings of the National Academy of Science USA 10.1073/pnas.2309536120. 10.1073/pnas.2309536120 PMC10576107 37782800

[CR36] Sassenrath-Cole GF, Pearcy RW (1994) Regulation of photosynthetic induction state by the magnitude and duration of low light exposure. Plant Physiol 105:1115–1123 12232269 10.1104/pp.105.4.1115 PMC159439

[CR37] Shao B, Zhang Y, Vincenzi E, Berman S, Vialet-Chabrand S, Marcelis LFM, Li T, Kaiser E (2024) Photosynthesis and photoprotection in top leaves respond faster to irradiance fluctuations than bottom leaves in a tomato canopy. J Exp Bot 75(22):7217–7236. 10.1093/jxb/erae357 39171726 10.1093/jxb/erae357 PMC11630027

[CR39] Sharkey TD, Seemann JR, Pearcy RW (1986) Contribution of metabolites of photosynthesis to postillumination CO_2_ assimilation in response to lightflecks. Plant Physiol 82:1063–1068 16665135 10.1104/pp.82.4.1063 PMC1056259

[CR38] Sharkey TD, Preiser AL, Weraduwage SM, Gog L (2020) Source of ^12^C in Calvin-Benson cycle intermediates and isoprene emitted from plant leaves fed with ^13^CO_2_. Biochem J 477(17):3237–3252. 10.1042/BCJ20200480 32815532 10.1042/BCJ20200480 PMC7666771

[CR40] Shimakawa G, Kohara A, Miyake C (2020) Characterization of light-enhanced respiration in cyanobacteria. Int J Mol Sci 22(1). 10.3390/ijms22010342 10.3390/ijms22010342 PMC7796093 33396191

[CR41] Stitt M (1986) Limitation of photosynthesis by carbon metabolism I. Evidence for excess electron transport capacity in leaves carrying out photosynthesis in saturating light and CO 2. Plant Physiol 81:1115–1122 16664953 10.1104/pp.81.4.1115 PMC1075495

[CR42] Stitt M, ap Rees T (1979) Capacities of pea chloroplasts to catalyse the oxidative pentose phosphate pathway and glycolysis. Phytochemistry 18:1905–1911

[CR44] Stitt M, Zhu XG (2014) The large pools of metabolites involved in intercellular metabolite shuttles in C_4_ photosynthesis provide enormous flexibility and robustness in a fluctuating light environment. Plant Cell Environ 37(9):1985–1988. 10.1111/pce.12290 24506493 10.1111/pce.12290

[CR43] Stitt M, Bulpin PV, ap Rees T (1978) Pathway of starch breakdown in photosynthetic tissues of Pisum sativum. Biochim Biophys Acta 544:200–214 152656 10.1016/0304-4165(78)90223-4

[CR45] Szecowka M, Heise R, Tohge T, Nunes-Nesi A, Vosloh D, Huege J, Feil R, Lunn J, Nikoloski Z, Stitt M, Fernie AR, Arrivault S (2013) Metabolic fluxes in an illuminated *Arabidopsis* rosette. Plant Cell 25:694–714. 10.1105/tpc.112.106989 23444331 10.1105/tpc.112.106989 PMC3608787

[CR46] Tanaka K, Shirai T, Vavricka CJ, Matsuda M, Kondo A, Hasunuma T (2022) Dark accumulation of downstream glycolytic intermediates initiates robust photosynthesis in cyanobacteria. Plant Physiol 191(4):2400–2413. 10.1093/plphys/kiac602 10.1093/plphys/kiac602 PMC10069908 36574371

[CR47] Tcherkez G, Abadie C, Dourmap C, Lalande J, Limami AM (2024) Leaf day respiration: more than just catabolic CO2 production in the light. Plant Cell Environ 47(7):2629–2637. 10.1111/pce.14904 10.1111/pce.14904 38528759

[CR48] Trefely S, Ashwell P, Snyder NW (2016) FluxFix: automatic isotopologue normalization for metabolic tracer analysis. BMC Bioinformatics 17(1):485. 10.1186/s12859-016-1360-7 27887574 10.1186/s12859-016-1360-7 PMC5123363

[CR49] Vickers CE, Possell M, Cojocariu CI, Velikova VB, Laothawornkitkul J, Ryan A, Mullineaux PM, Hewitt CN (2009) Isoprene synthesis protects Transgenic tobacco plants from oxidative stress. Plant Cell Environ 32:520–531. 10.1111/j.1365-3040.2009.01946.x 19183288 10.1111/j.1365-3040.2009.01946.x

[CR50] Voon CP, Lim BL (2019) ATP translocation and Chloroplast biology. Natl Sci Rev 6(6):1073–1076. 10.1093/nsr/nwz089 34691977 10.1093/nsr/nwz089 PMC8291404

[CR51] Wulfert S, Schilasky S, Krueger S (2020) Transcriptional and biochemical characterization of cytosolic pyruvate kinases in *Arabidopsis Thaliana*. Plants 9(3):353 32168758 10.3390/plants9030353 PMC7154858

[CR52] Xu Y, Fu X, Sharkey TD, Shachar-Hill Y, Walker B (2021) The metabolic origins of non-photorespiratory CO_2_ release during photosynthesis: A metabolic flux analysis. Plant Physiol 186:297–314. 10.1093/plphys/kiab076 33591309 10.1093/plphys/kiab076 PMC8154043

[CR55] Xu Y, Wieloch T, Kaste JAM, Shachar-Hill Y, Sharkey TD (2022) Reimport of carbon from cytosolic and vacuolar sugar pools into the Calvin-Benson cycle explains photosynthesis labeling anomalies. Proceedings of the National Academy of Sciences 119 (11):e2121531119. doi:10.1073/pnas.2121531119 10.1073/pnas.2121531119 PMC8931376 35259011

[CR54] Xu Y, Schmiege SC, Sharkey TD (2024) The oxidative pentose phosphate pathway in photosynthesis: a tale of two shunts. New Phytol 242(6):2453–2463. 10.1111/nph.19730 38567702 10.1111/nph.19730

[CR53] Xu Y, Kaste J, Weise S, Shachar-Hill Y, Sharkey T (2025) The effects of photosynthetic rate on respiration in light, starch/sucrose partitioning, and other metabolic fluxes within photosynthesis. Sci Rep 15:8389. 10.1038/s41598-025-88574-4 40069307 10.1038/s41598-025-88574-4 PMC11897357

[CR56] Zimmermann SE, Benstein RM, Flores-Tornero M, Blau S, Anoman AD, Rosa-Téllez S, Gerlich SC, Salem MA, Alseekh S, Kopriva S, Wewer V, Flügge UI, Jacoby RP, Fernie AR, Giavalisco P, Ros R, Krueger S (2021) The phosphorylated pathway of Serine biosynthesis links plant growth with nitrogen metabolism. Plant Physiol 186(3):1487–1506. 10.1093/plphys/kiab167 34624108 10.1093/plphys/kiab167 PMC8260141

